# Double-Level Energy Absorption of 3D Printed TPMS Cellular Structures via Wall Thickness Gradient Design

**DOI:** 10.3390/ma14216262

**Published:** 2021-10-21

**Authors:** Minting Zhong, Wei Zhou, Huifeng Xi, Yingjing Liang, Zhigang Wu

**Affiliations:** 1 School of Civil Engineering, Guangzhou University, Guangzhou 510006, China; 2111916048@e.gzhu.edu.cn (M.Z.); z18826737435@163.com (W.Z.); yjliang@gzhu.edu.cn (Y.L.); 2 MOE Key Laboratory of Disaster Forecast and Control in Engineering, School of Mechanics and Construction Engineering, Jinan University, Guangzhou 510632, China; xihuifeng@jnu.edu.cn

**Keywords:** triply periodic minimal surface, selective laser melting, 316 L stainless steel, energy absorption, deformation mechanism

## Abstract

This paper investigates the deformation mechanism and energy absorption behaviour of 316 L triply periodic minimal surface (TPMS) structures with uniform and graded wall thicknesses fabricated by the selective laser melting technique. The uniform P-surface TPMS structure presents a single-level stress plateau for energy absorption and a localized diagonal shear cell failure. A graded strategy was employed to break such localized geometrical deformation to improve the overall energy absorption and to provide a double-level function. Two segments with different wall thicknesses separated by a barrier layer were designed along the compression direction while keeping the same relative density as the uniform structure. The results show that the crushing of the cells of the graded P-surface TPMS structure occurs first within the thin segment and then propagates to the thick segment. The stress–strain response shows apparent double stress plateaus. The stress level and length of each plateau can be adjusted by changing the wall thickness and position of the barrier layer between the two segments. The total energy absorption of the gradient TPMS structure was also found slightly higher than that of the uniform TPMS counterparts. The gradient design of TPMS structures may find applications where the energy absorption requires a double-level feature or a warning function.

## 1. Introduction

Porous metallic materials have been extensively researched, which can be attributed to their extraordinary properties, such as their high strength to weight ratio, excellent thermal insulation, and damping properties [1,2,3], which may find useful applications in bone implants [4], heat exchangers [5], and energy absorption [6]. Some popular porous structures are largely inspired by topologies that found in nature, such as body-centered cubic (BCC) [7], rhombic dodecahedron [8], honeycomb structures [9], and truss–lattice structures [10,11], which show geometry periodicity and topological homogeneity. However, these structures commonly suffer nodal stress concentration and may be subject to instable mechanical performance and premature failure [12,13,14].

Recently, the nature-inspired triply periodic minimal surface (TPMS) structures that have been found in urchins [15], butterfly wing scales [16], and the exoskeletons of beetles [17] have attracted a great deal of attention. TPMSs are mathematically rigorous, with a zero–mean curvature, and are composed of infinite and non-self-intersecting surfaces that are associated with a specific crystallographic space group symmetry [18]. A TPMS has a long-range ordered structure with a high specific surface area, high porosity, and stable mechanical properties. Its internal structure is interconnected, and its surface is smooth. These appealing properties of TPMS structures promotes uniform stress distribution on the surface of cell walls and avoids the stress concentration under loading. Hence, it is an ideal porous structure for structural energy absorption design.

Investigations on the structure design and mechanical performance of metallic TPMS structures have been conducted extensively since the emergence of the additive manufacturing (AM) technique. The compressive deformation characteristics of TPMS structures are similar to those of conventional metallic foams, showing three distinctive deformation stages, including an initial linear elastic stage, a middle stress plateau stage, and a final densification stage [19,20]. The energy absorption capability is mainly contributed from the plateau stage, and this normally requires a stable stress level and long plateau strain. Zhang et al. reported that the TPMS structures fabricated by selective laser melting (SLM) possess superior stiffness, plateau stress, and energy absorption ability relative to the BCC lattice [21]. Liang et al. also fabricated 316 L TPMS structures with different relative densities by SLM. They found that deformation of the cells concentrates much along the diagonal band regions, and when the relative density was lower than 0.35, the yield strength and energy absorption capacity of the P-surface were better than those of the G-surface [22]. Yang et al. studied the fatigue properties of G-surface TPMS structures and found the failure samples have nearly 45° fracture bands along the diagonal surface [23]. It can be seen that the stress concentration and the dominant diagonal shear failure of the uniform TPMS structures limits their energy absorption performance.

As a type of important porous structure, functionally graded structures are the optimal choice to tackle the issue of stress concentration and are promising for creating light weight and high energy absorption parts. The properties of functionally graded structures include the gradient wall thickness, pore size, and unit cell type along the loading direction. Fan et al. proposed that a graded wall thickness design can effectively improve the mechanical properties of the structure. Compared with the uniform thickness structure, the elastic modulus of the graded sample is increased by more than 94 MPa, and the cumulative energy absorption is increased by 15.4% [24]. Min et al. simulated the in-plane impact properties and energy absorption properties of concave hexagonal honeycomb materials with a gradient cell thickness [25]. They found that the concave hexagonal honeycomb materials with a positive thickness gradient have better energy absorption and impact resistance than the uniform structure. Al-Ketan et al. conducted mechanical tests on gradient samples in parallel and perpendicular to the grading direction to study the effect of the loading direction on the displayed deformation mechanism [26]. When the load is applied parallel to the grading direction, the collapse starts layer by layer, and the deformed layer densifies at the top of the next one, resulting in a hardening behavior. The test perpendicular to the grading direction showed a deformation mode along the diagonal shear band that was similar to the uniform structure. Yu et al. showed that the stress in the wall thickness-graded Schwarz P-surface structure gradually exceeds that in the uniform P-surface structure and that the total absorbed energy per unit volume W_vD_ is 1.5 times higher than that of the uniform P-surface structure [27]. Panesar et al. evaluated the mechanical properties of the proposed gradient thickness design and showed that the intersecting/grading/scaling lattice strategy is 40–50% better than the uniform structure in terms of specific stiffness [28].

To summarize the above literature findings, one can see that the gradient TPMS structure has great potential to achieve high mechanical performance and to improve the energy absorption ability of the structure. The gradient designs in the previous reports normally follow a linear grading of properties (wall thickness, lattice type, and cell size gradient) that is parallel to the loading direction. The middle stress plateau evolves into to a gradual strain hardening stage following the implementation of gradient design. In this study, we propose a different gradient design in the wall thickness. The distribution of different wall thickness is segmental and discrete in order to break the original localized deformation within the diagonal shear bands. Such a design provides a characteristic double-stage energy absorption characteristic during the plastic deformation of the structure. This may shed a light on novel gradient design for applications requiring multi-level energy absorption.

## 2. Materials and Methods

### 2.1. TPMS Models

The primitive P-surface TPMS structure was selected to study the gradient design and its effect on the deformation mechanism. The approximate value of the P-surface is defined by the below formula [29]:

(1) $$\Phi_{P}\left(x,y,z\right)=cos\left(\omega x\right)+cos\left(\omega y\right)+\cos\left(\omega z\right)=c$$

where *Φ_p_* is a surface calculated at the isovalue *c*, ω=2π/L, and *L* is the length of the unit cell. The expansion of the surface in three-dimensional space can be controlled by modifying *c*. When *c* = 0, the resulting surfaces divide the space into sub-domains of equal volumes. Then, the structure of TPMS is realized by surface thickening into the form of a continuous shell with limited thickness [30]. The P-surface TPMS model has a unit cell length of 4 mm and an array of 8 × 8 × 8 tessellating cells. The TPMS structure with different wall thicknesses is obtained by using the plug-in Grasshopper in Rhino, and then the STL file is generated.

### 2.2. Gradient Design

**Figure 1:**
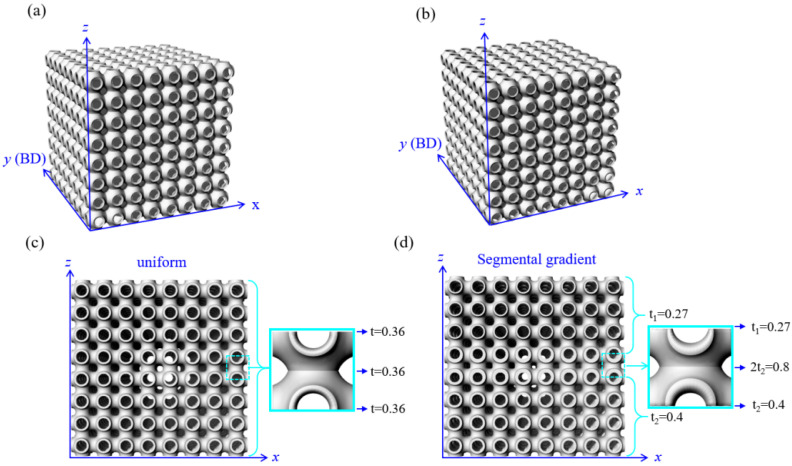
Structure models of the uniform and gradient TPMS structures. (**a**) Model of Uni-1, (**b**) model of Gra-1, (**c**) the front view of Uni-1, and (**d**) the front view of Gra-1 showing the gradient thickness distribution along *z*-axis. BD indicates building direction. BD ⊥ Gradient direction. The values labelled on the models are in mm.

Uniform and gradient P-surface TPMS structures were investigated. Figure 1 shows the comparison between the uniform (Uni-1) and gradient (Gra-1) models, which had a similar relative density of ~0.22. Figure 1a,b show the three-dimensional view of the uniform and gradient models, respectively. Figure 1c,d present the front face (*x*-*z* plane) of the models and shows the details of the wall thickness arrangement. Uni-1 apparently has a constant wall thickness of t = 0.36 mm throughout the model. In contrast, the wall thickness of Gra-1 distributes differently along the *z*-axis (loading direction). The top segment, which includes 1–3.5 layers, is relatively thinner, with a wall thickness of t_1_ = 0.27 mm, while the bottom segment, which includes 4.5–8 layers, is thicker, with a wall thickness of t_2_ = 0.4 mm. A transition or barrier layer is introduced between these two segments, covering the 3.5–4.5 layer. The gradient thickness of this barrier layer follows t_1_→2t_2_→t_2_, which is 0.27→1.08→0.54 mm in the case of Gra-1. The purpose of having 2t_2_ in the middle of the barrier layer is to introduce a dense layer to better separate the thin and thick segments under loading, i.e., to make a sharp stepping feature of the double stress plateaus. The sample ID, wall thickness, mass, relative density, and wall thickness distribution of all of the samples are detailed in Table 1.

### 2.3. Finite Element Analysis

The single cell model of the P-surface was first generated by the mathematical formula (Equation (1)) in Rhino software (version 6.23) and was then saved as an igs format file and imported to the Hypermesh software (version 14) for mesh distribution. Since the wall thickness of the TPMS model is much smaller than the size of the model, shell element (S4R) was used for the construction of the model. Abaqus (version 6.14-1 under academic license) was used to simulate the compressive behavior of the TPMS models. The meshed unit cell of the P-surface was imported into Abaqus, and the array function was used to form an 8 × 8 × 8 model. The base material, 316 L stainless steel, was modelled using an elastic model and a simple rate-independent J2 plastic model with isotropic hardening in Abaqus. The material properties of 316 L stainless steel defined in Abaqus are given in Table 2. The wall thicknesses of the FE models were set to keep their relative densities identical to those of the tested samples. Two steel plates with infinite stiffness were established using a discrete rigid body. The displacement control was implemented to the top steel plate, with a strain rate that was the same as the compression experiments, while all the degrees of freedom of the bottom steel plate were fixed.

### 2.4. SLM Fabrication

Stainless steel 316 L gas-atomized powder was provided by AMC Powder with an average particle size of 45 μm (D50). Table 3 shows the chemical composition of SS316 L powder. The TPMS samples were fabricated in 99.99% argon using an HBD-100 machine (Guangdong Hanbang 3D Technology Co.,Ltd, Zhongshan, China). A laser power (*P*) of 160 W, scanning speed (*v*) of 1000 mm/s, hatch spacing (*h*) of 80 μm, and layer thickness (*t*) of 30 μm were used in this study. According to *E* = *P*/(*v* × *h* × *t*), the delivered energy density (*E*) during the laser melting process can be calculated as 66.7 J/mm^3^. A bidirectional orthogonal scanning strategy was adopted for each layer.

### 2.5. Characterization Techniques

The surface morphology of the TPMS samples was examined using a scanning electron microscope (SEM, Japan Electronics Co., Ltd, Tokyo, Japan) and a sec JSM-7001F instrument (Japan Electronics Co., Ltd., Tokyo, Japan). The internal defects were checked using a nanoVoxel-3000 micro-CT instrument (Sanying Precision Instrument Co., Ltd, Tianjin, China). The quasi-static compression test of the TPMS samples was conducted using an ETM series electronic universal testing machine (Shenzhen SUNS Technology Stock Co., Ltd., Shenzhen, China) with a load capacity of 300 kN. The crosshead speed of 2 mm/min was used for testing. The compression direction was perpendicular to the building direction.

## 3. Results and Discussion

**Figure 2:**
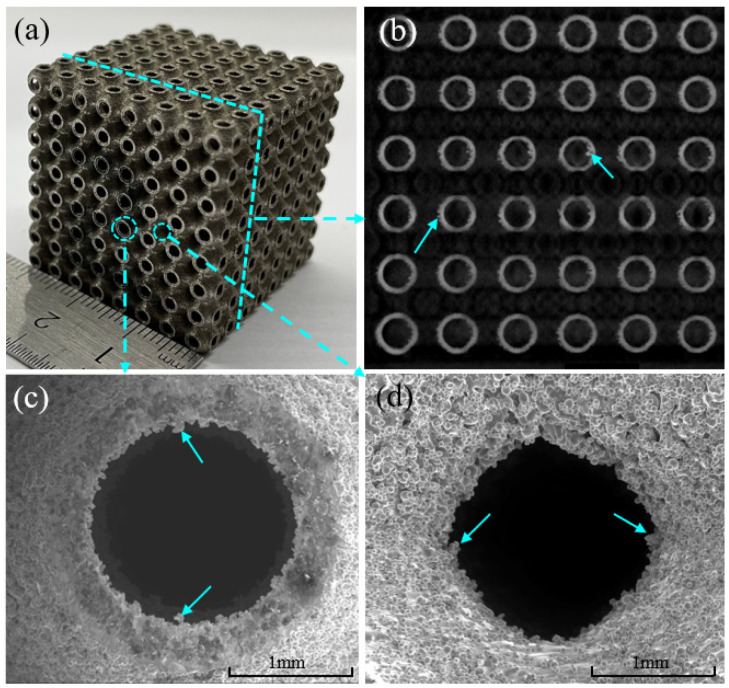
Printing geometry and surface quality of Uni-1. (**a**) A photograph of the overall appearance, (**b**) one of the CT scan images showing internal surface contour, and (**c**,**d**) SEM micrographs of selected locations (circled in (**a**)) on the x-z plane showing the presence of excessive residual particles.

Figure 2 shows the surface quality of the as-fabricated Uni-1. Figure 2a is a photograph of Uni-1, and Figure 2b is a representative CT scan image of the x-z plane at the location indicated by the dashed lines. It can be seen that the surface contours show minimal geometrical defects. The internal cross-section of the sample presents a continuous connection of the shell walls without sharp horns or irregularities. However, there are some locations showing particle adhesion on the inner surface of the TPMS structure (arrowed). Figure 2c,d are the SEM micrographs at different locations of the sample, as circled in (a). It should be noted that the surface is covered with numerous partially melted 316 L particles, which is a common surface imperfection in powder-bed AM techniques. In general, no gross defects such as holes or cracks were found within the as-fabricated samples.

**Figure 3:**
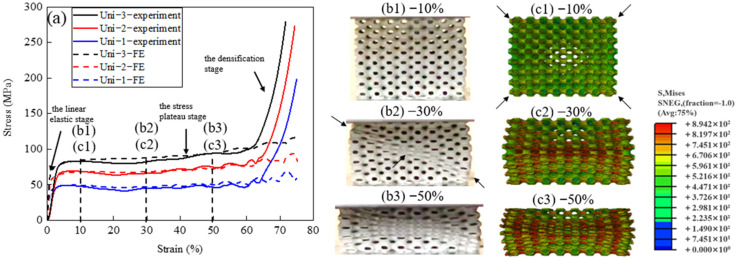
Deformation behaviour of uniform TPMS structures. (**a**) Stress–strain curves of Uni−1, Uni−2, and Uni−3, (**b1**–**b3**) snapshots of the side surface during compression experiment, and (**c1**–**c3**) Mises stress cloud diagrams of the side surface during FE simulation of Uni−1.

Figure 3 shows the compressive properties and deforming characteristics of the uniform TPMS samples. Figure 3a displays the compressive stress–strain curves (solid lines) and the corresponding FE stress–strain curves (dashed lines) of Uni-1, Uni-2, and Uni-3. The stress–strain curves can be divided into three distinct deformation stages: (1) 0–3%: the linear elastic stage, (2) 3–65%: the stress plateau stage, and (3) >65%: the densification stage. Stress fluctuations can be observed on the stress plateau when the applied strain is greater than 40%. Such mechanical instability of the P-surface TPMS structure has also been reported by other authors, which is due to the localized bulking of the thin and curved shell walls [21]. The experimental results show a relatively shorter stress plateau and an earlier onset of the densification compared to the FE results for all the three samples. This is because the actual average wall thicknesses in the as-fabricated samples are slightly greater than the designed values, as seen in Table 1, which caused the early self-contacting of the shell walls.

As the wall thickness increases (Uni−1→Uni−2→Uni−3), the yield strength increases progressively from 47 MPa to 83 Mpa. In the meantime, the length of stress plateau shortens slightly, and the stress fluctuation phenomenon weakens. This implies better mechanical stability in structures with greater wall thicknesses. Figure 3(b1–b3,c1–c3) are the snapshots of the side surface (x-z plane) of Uni-1 at different applied strains during the experiment and the corresponding FE Mises stress cloud diagrams during the simulation. The snapshots and Mises stress cloud diagrams are selected at 10%, 30% and 50% of deformation, as labeled in (a). Generally speaking, the cells in uniform P-surface collapse in layers and show a localized double shear band geometry on the side surface during the experiment, which is consistent with the deformation in the FE simulation. When the strain is 10%, the cell openings of the four corners show the largest stress magnitude (arrowed) and thus the largest cell deformation. At 30%, the local deformation pattern shows severe cell crushing along only one diagonal shear band in the experiment, while the model in the FE simulation shows a symmetric double shear band stress concentration and cell deformation. The asymmetrical deformation pattern is likely due to the uneven defect distribution in this sample. When the strain reached 50%, the cells were squeezed into symmetrical double shear bands in the experiment, and only the cells outside of the shear band geometry were not completely closed, as shown in both the experimental and simulated results.

**Figure 4:**
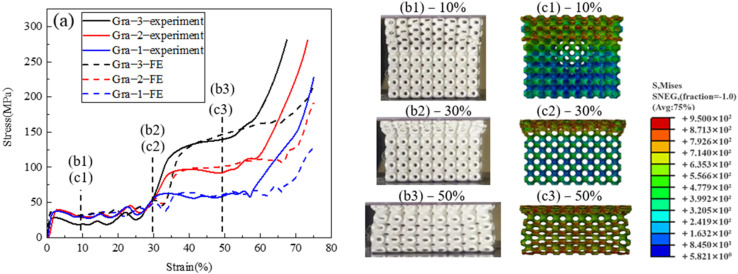
Deformation behaviour of the gradient TPMS structures with different heights of the second stress plateau. (**a**) stress–strain curves of Gra−1, Gra−2, and Gra−3 and (**b1**–**b3**) snapshots of Gra−3 at 10%, 30%, and 50% strain during the compression experiment. (**c1**–**c3**) Mises stress cloud diagrams of Gra−3 at 10%, 30%, and 50% strain during the FE simulation.

Figure 4 shows the mechanical properties of Gra−1, Gra−2, and Gra−3 gradient structures. In this group, the wall thickness keeps constant at t_1_ = 0.27 mm from 1–3.5 layers along the z-direction for all three samples, while the wall thickness t_2_ of 4.5–8 layers is thicker, being 0.4 mm, 0.54 mm, and 0.67 mm for Gra−1, Gra−2, and Gra−3, respectively. This gives rise to a thinner upper segment and thicker lower segment in these samples. The middle section of 3.5–4.5 layer is a wall thickness transition section and also acts as a barrier layer to separate the deformation of the thick segment from the thin one since the thickness gradient is designed as t_1_→2t_2_→t_2_.

Figure 4a shows the stress–strain curves of Gra−1, Gra−2, and Gra−3, in which the solid lines are the experimental results and where the dashed lines are the FE simulation results. It can be seen that the gradient structures clearly present two distinct stress plateaus before and after 30% of the deformation strain. The first stress plateau continues to be almost unchanged at the lower stress level of ~40 MPa with the presence of some minor stress fluctuations, while the second stress plateau is higher and rises progressively from 60–140 MPa with the increase of t_2_. In general, the FE simulation stress–strain responses show good agreement with the experimental results. Small discrepancies exist in the onset of the second stress plateau and in the densification between the experimental and simulation results. This is because the real wall thicknesses of the printed samples are slightly greater than the designed values, thus showing earlier self-contacting of the collapsed cell walls. Compared to the uniform structures (Figure 3a), the densification stage of the gradient structures appears to be less clearly presented, as gradual hardening is shown first and is then followed by the rapidly rising stress–strain slope. This is due to the gradual deformation of the barrier layer at the end of the deformation plateau and occurs simultaneously with the densification process of the 1–3.5 and 4.5–8 layers.

Figure 4(b1–b3,c1–c3) show the experimental snapshots and FE Mises stress diagrams at different applied strains on the side surface of Gra-3. When the structure is deformed to 10%, heavy deformation of the open cells can be seen within the upper thin segment, i.e., 1–3.5 layers. Close inspection on the side surface reveals a double diagonal deformation within the thin section (Figure 4(b1)). The corresponding FE Mises stress map (Figure 4(c1)) also shows the localized stress distribution along the double shear band. When the applied strain reaches 30%, the thin section is almost fully collapsed, while the cells in the lower thick section show minimal changes in shape (Figure 4(b2)). At the same time, the FE Mises stress map shows large stress values within the squeezed upper segment, and small values within the lower segment (Figure 4(c2)). When the structure is deformed to 50%, the thick segment shows clear crushed cells as deformation enters the middle of the second stress plateau (Figure 4(b3)). The corresponding FE Mises stress cloud map (Figure 4(c3)) shows a similar cell deformation appearance, and a second pair of double shear bands with high stress magnitudes also appear.

**Figure 5:**
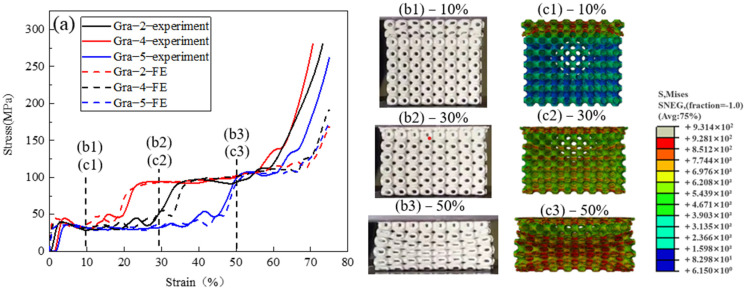
Deformation behaviour of the gradient TPMS structures with different lengths of the second stress plateau. (**a**) stress–strain curves of Gra−2, Gra−4, and Gra−5 samples, (**b1**–**b3**) snapshots of Gra−4 at 10%, 30%, and 50% strain during the compression experiment, and (**c1**–**c3**) Mises stress cloud diagrams of Gra−4 at 10%, 30%, and 50% strain during the FE simulation.

Figure 5 shows the deformation behaviour of another group of gradient samples, Gra−2, Gra−4, and Gra−5. In this group, t_1_ and t_2_ are kept constant at 0.27 and 0.54 mm, while the position of the barrier layer (0.27–1.08–0.54 mm) varies. To be specific, the barrier layer is located at the 3.5–4.5, 2–3, and 5–6 layer for Gra−2, Gra−4, and Gra−5, respectively. The simulation results are well in line with the experimental results. The main difference among the three samples is the change of plateau length. Figure 5(b1–b3,c1–c3) show the experimental snapshots and FE simulated Mises stress diagrams at different applied strains on the side surface of Gra−4 during compression. It can be seen that the localized deformation occurs sequentially from the thin segment to the thick segment separated by the barrier layer.

**Figure 6:**
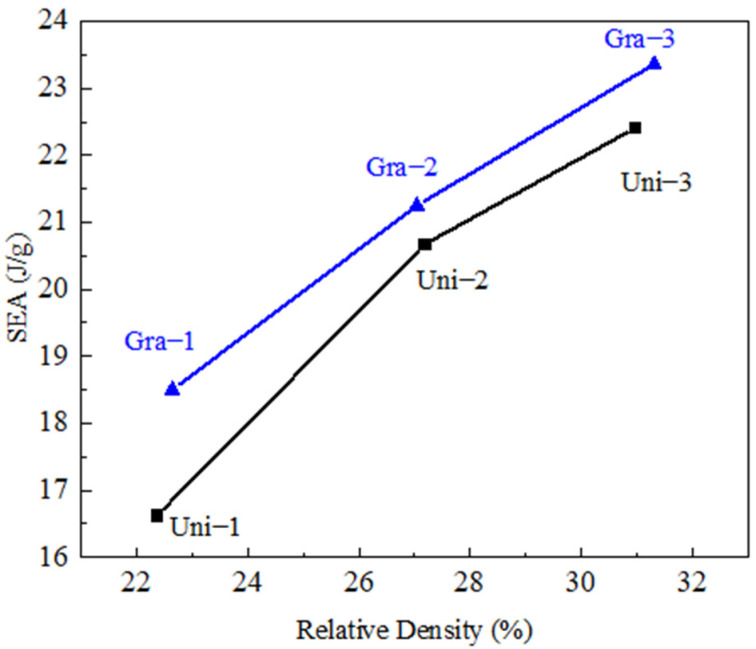
SEA comparison of the uniform and gradient TPMS structures with similar relative densities.

The specific energy absorption (SEA) is a meaningful engineering parameter to evaluate the energy absorption characteristics of a structure. It is defined as $\mathrm{SEA}=\frac{W}{\rho }$, where *W* is the energy absorbed under compression at a given strain and where *ρ* is the mass density of the cellular structure. In this study, *W* was obtained by integrating the stress–strain curves up to a deformation strain of 65%, which is the onset of densification of the uniform structures. In general, the energy absorption values of the P-surface with gradient design slightly outperformed the uniform counterparts by 3–9%, as seen in Figure 6. This is because the segmental gradient design of the wall thickness breaks the localized deformation within the original double shear bands of the uniform structure and distributes the plastic deformation of the entire structure more sufficiently and evenly. The separation of the two segments with different wall thickness (relative density) improves the overall effectiveness of the cell deformation in the structure, thus improving the energy absorption performance.

## 4. Conclusions

In this study, uniform and gradient P-surface TPMS structures with different relative densities were designed and fabricated using the SLM technique. The deformation mechanism and energy absorption behaviour of the structures were investigated, which led to the following conclusions:

1. The as-fabricated TPMS samples show overall satisfactory geometrical and surface quality with freedom from any gross internal or external defects. The presence of residual 316 L particles on the curved surfaces contributed to a rough surface outline as well as a slightly greater sample mass relative to the designed values.
2. The uniform TPMS structures show a typical localized deformation pattern within a double shear band geometry. The yield stress and plateau stress increase with increasing the relative density.
3. The gradient TPMS samples show a double-leveled deformation manner with a two stress plateaus on the stress–strain responses. The height and length ratio between the stress plateaus can be adjusted by changing the wall thickness in each segment and by adjusting the position of the barrier layer in the structure.
4. The specific energy absorption SEA of the gradient TPMS structures are slightly higher than the uniform counterparts with similar relative densities.

## Figures and Tables

**Table 1:** Sample ID, sample mass, relative density, and wall thickness distribution of the designed uniform and gradient TPMS structures.

Wall Thickness Type	Sample ID	Designed Mass (g)	Actual Mass (g)	Designed Relative Density (%)	Actual Relative Density (%)	Wall Thickness Distribution along *z*-axis (mm)
**Uniform**	Uni-1	55.87	58.45	21.37	22.36	1–8 layers: t = 0.36
	Uni-2	68.63	71.08	26.24	27.18	1–8 layers: t = 0.45
	Uni-3	80.47	80.99	30.77	30.97	1–8 layers: t = 0.52
**Gradient**	Gra-1	55.87	59.19	21.37	22.64	1–3.5 layers: t_1_: 0.27 3.5–4.5 layer: t_1_-2t_2_-t_2_: 0.27–0.8–0.4 4.5–8 layers: t_2_: 0.4
	Gra-2	68.63	70.71	26.24	27.04	1–3.5 layers: t_1_: 0.27 3.5–4.5 layers: t_1_-2t_2_-t_2_: 0.27–1.08–0.54 4.5–8 layers: t_2_: 0.54
	Gra-3	80.47	81.86	30.77	31.31	1–3.5 layers: t_1_: 0.27 3.5–4.5 layer: t_1_-2t_2_-t_2_: 0.27–1.34–0.67 4.5–8 layers: t_2_: 0.67
	Gra-4	76.39	77.32	29.22	29.58	1–2 layers: t_1_: 0.27 2–3 layer: t_1_-2t_2_-t_2_: 0.27–1.08–0.54 3–8 layers: t_2_: 0.54
	Gra-5	60.86	63.96	23.27	24.46	1–5 layers: t_1_: 0.27 5–6 layer: t_1_-2t_2_-t_2_: 0.27–1.08–0.54 6–8 layers: t_2_: 0.54

**Table 2:** Material parameters of the base material 316 L used in FE simulation.

Material Density (g/cm^3^)	Elasticity Type	Yield Strength (MPa)	Young’s Modulus (MPa)	Poisson’s Ratio	Plasticity Type	Hardening Rule
7.98	Isotropic	460	169,000	0.3	Von-Mises yield criterion	Isotropic

**Table 3:** Chemical composition of 316 L powder.

Element	Cr	Ni	Mo	Mn	Si	Cu	Fe
wt.%	17.35	12.02	2.74	1.36	0.33	0.23	bal.

## Data Availability

The raw data required to reproduce these findings are available from the corresponding author by request.
